# Assessment of Sarcopenia in Patients with Liver Cirrhosis—A Literature Review

**DOI:** 10.3390/nu17162589

**Published:** 2025-08-09

**Authors:** Dorotea Bozic, Bisera Mamic, Iva Peric, Ivona Bozic, Ivan Zaja, Tomislav Ivanovic, Ana Gugic Ratkovic, Ivica Grgurevic

**Affiliations:** 1 Department of Gastroenterology, University Hospital of Split, Spinciceva 1, 21000 Split, Croatia; ivan.zaja@yahoo.com; 2 Department of Oncology and Radiotherapy, University Hospital of Split, Spinciceva 1, 21000 Split, Croatia; bisera208@gmail.com; 3 Department of Radiology and Interventional Radiology, University Hospital of Split, Spinciceva 1, 21000 Split, Croatia; ivaperic@kbsplit.hr; 4 Department of Rheumatology and Immunology, University Hospital of Split, Soltanska 2, 21000 Split, Croatia; ivona.bozic.7@gmail.com; 5 University Department of Health Studies, University of Split, Rudjera Boskovica 35, 21000 Split, Croatia; 6 Department of Abdominal Surgery, University Hospital of Split, Spinciceva 1, 21000 Split, Croatia; tomo.mefst@gmail.com; 7 Faculty of Food Technology and Biotechnology, University of Zagreb, Pierrotijeva 6, 10000 Zagreb, Croatia; ana.gugic.ratkovic@gmail.com; 8 Department of Gastroenterology, Hepatology and Clinical Nutrition, Clinical Hospital Dubrava, Avenija Gojka Suska 6, 10000 Zagreb, Croatia; ivicag72@gmail.com; 9 School of Medicine, University of Zagreb, Salata 3, 10000 Zagreb, Croatia; 10 Faculty of Pharmacy and Biochemistry, University of Zagreb, Ante Kovacica 1, 10000 Zagreb, Croatia

**Keywords:** sarcopenia, liver cirrhosis, L3-SMI, BIA, muscle thickness

## Abstract

Sarcopenia refers to a disorder involving the gradual and overall reduction in skeletal muscle mass and physical capability. It occurs in over one-third of individuals with liver cirrhosis and serves as an independent predictor of increased mortality risk. Assessment of sarcopenia is necessary in all patients with liver cirrhosis, as recommended by the European Association for the Study of the Liver (EASL) and the European Society for Clinical Nutrition and Metabolism (ESPEN). The evaluation of muscle mass can be performed using several validated methods such as the multislice computed tomography (MSCT), abdominal magnetic resonance imaging (MRI), dual X-ray absorptiometry (DXA), bioelectrical impedance analysis (BIA), or muscle ultrasound. Assessment of muscle function encompasses measurements of both muscle strength and physical performance. Sarcopenia has a significant negative impact on the course of the disease, quality of life and outcomes of patients with liver cirrhosis. Considering the global healthcare impact and the significant influence on the course of disease, characteristics of simplicity, swiftness, safety, availability, reproducibility, and diagnostic accuracy are certainly the key factors to consider when choosing the proper diagnostic method for nutritional assessment. The aim of this review is to analyze the pathophysiological mechanisms underlying muscle mass loss in patients with liver cirrhosis, as well as to assess strengths and limitations of the methods currently in use to diagnose sarcopenia.

## 1. Introduction

Liver cirrhosis is an end-stage liver disease, considered to be the cause of more than 1.32 million deaths per year [1,2]. Sarcopenia (sarko (meat, from Greek σαρκο) and penia (loss, from Greek πενία)) is a muscle disorder associated with aging, characterized by the progressive loss of muscle mass, strength, and functional capacity. The decline in muscular mass and strength observed in chronic conditions—such as hepatic cirrhosis—may also be classified as a disease-related sarcopenia, which will be examined in the present review [3,4,5].

Sarcopenia occurs in over one-third of individuals with liver cirrhosis and serves as an independent predictor of increased mortality risk [6,7]. Its prevalence has been estimated at 37.5% but reaches up to 42% in men and up to 50% in patients with alcoholic liver disease (ALD) or decompensated cirrhosis [6].

The aim of this review is to analyze the pathophysiological mechanisms that contribute to muscle mass loss and to evaluate the advantages and disadvantages of the available armamentarium of methods in the diagnosis of sarcopenia in patients with liver cirrhosis.

The relevant literature was searched using the PubMed database, concluding on 1 May 2025. We searched using the combination of the following MeSH terms: “liver cirrhosis”, “sarcopenia”, “L3-SMI”, “BIA”, “dual X-ray absorptiometry”, “magnetic resonance imaging”, “MSCT psoas muscle”, “pathophysiology”, “screening”, “muscle mass”, “muscle strength”, “muscle ultrasound”, and “anthropometric methods”.

## 2. Pathophysiology of Sarcopenia in Liver Cirrhosis

Sarcopenia develops as a consequence of reduced physical activity, inadequate diet, malabsorption, alcohol consumption, low testosterone levels, systemic inflammation, high myostatin levels, low glycogen stores, and excessive protein use as an energy source [8]. Appetite regulation involves a complex neuroendocrine system, with ghrelin as the primary orexigenic hormone and several anorexigenic hormones, including leptin. In cirrhosis, patients typically exhibit reduced ghrelin levels, elevated leptin levels, and impaired satiety signaling, contributing to anorexia, which is further exacerbated by proinflammatory cytokines. Additionally, hyperdynamic circulation and sympathetic overactivity increase energy expenditure, further worsening nutritional status [9]. Malabsorption occurs on the basis of intestinal bacterial overgrowth with subsequent bile acid deconjugation, intestinal mucosal edema, and portal hypertension-induced enteropathy [10]. Dysbiosis contributes to systemic inflammation by increasing the abundance of pro-inflammatory and reducing the abundance of anti-inflammatory gut bacteria. Additionally, dysbiosis disrupts amino acid metabolism and facilitates the translocation of endotoxins, thereby exacerbating inflammation, liver dysfunction, and muscle wasting [11].

In addition to the direct testicular injury caused by ethanol, individuals with liver cirrhosis suffer from central hypogonadism, resulting in low testosterone, luteinizing hormone (LH), and hepatocyte growth factor alpha (HGF-α) levels [12].

Systemic inflammation in cirrhosis is caused by high levels of DAMPs (damage-associated molecular patterns), which are released as a result of hepatocyte necrosis, and PAMPs (pathogen-associated molecular patterns), which are released as a consequence of bacterial translocation from the intestinal lumen into the bloodstream [13]. Inflammatory cytokines further lead to reduced protein synthesis via myostatin, which stimulates protein catabolism and inhibits myogenic stem cells. It is compelling that elevated myostatin levels are also conditioned by elevated ammonia values [10].

Qiu et al. demonstrated that hyperammonemia triggers the activation of IkappaB (IκB) kinase, promotes nuclear translocation of nuclear factor kappa B (NF-κB), facilitates the binding of the NF-κB p65 subunit to specific regions of the myostatin promoter, and ultimately induces transcription of the myostatin gene [14]. Insulin growth factor 1 (IGF1) is important for protein anabolism by stimulating the mammalian target of the rapamycin (mTOR) signaling pathway and simultaneously inhibiting the synthesis of myostatin. In patients with a cirrhotic liver, low values of growth factors such as IGF-1 and decreased activity of the anabolic mTOR signaling pathway were recorded, while on the other hand, the activity of catabolic signaling pathways such as the ubiquitin–proteasome (U-P) system, which is affected by insulin and testosterone levels, starvation, and metabolic acidosis, were increased [10,15]. From a pathophysiological perspective, the primary driver of enhanced protein catabolism in cirrhotic patients is the depletion of hepatic glycogen reserves, which leads to accelerated fat and protein catabolism. The main sources of gluconeogenesis are glycogen stores and branched-chain amino acids (BCAA; i.e., 3 essential amino acids: leucine, isoleucine, and valine) from the muscle tissue [10,15].

## 3. Definition and Screening

Sarcopenia assessment is necessary in all patients with liver cirrhosis, as recommended by the European Association for the Study of the Liver (EASL) and the European Society for Clinical Nutrition and Metabolism (ESPEN) [8,16]. The currently accepted definitions of sarcopenia are presented in Table 1.

**Scheme 1:**
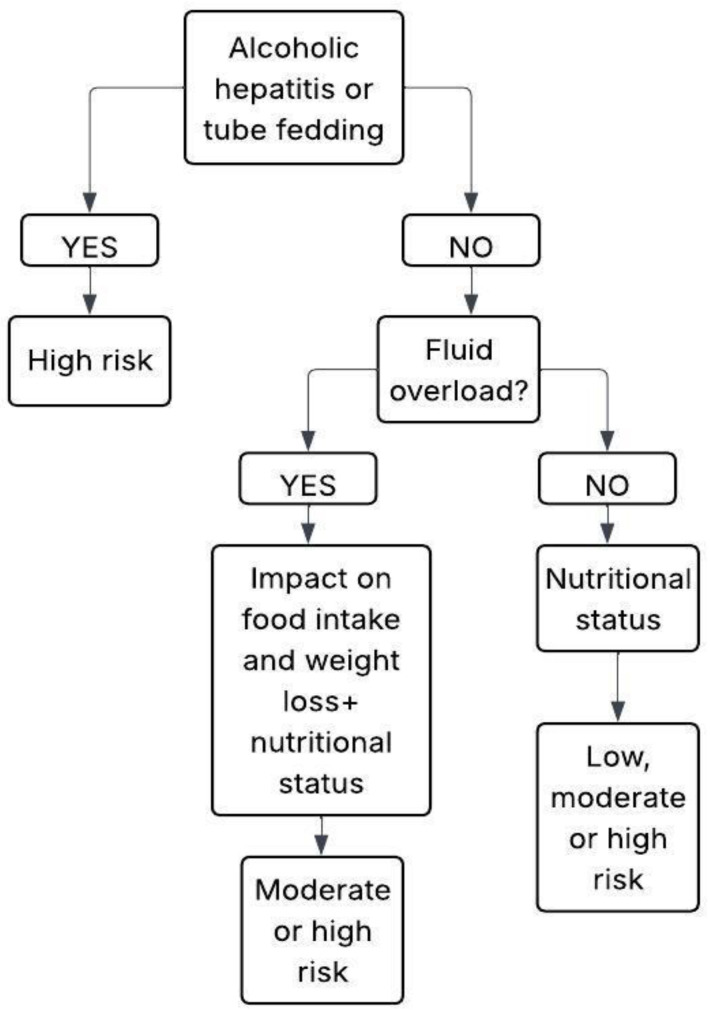
A simplified Royal Free Hospital Nutritional Prioritizing Tool.

The EASL recommends assessing the nutritional status of all patients with a body mass index (BMI) of 18.5–29.9 kg/m^2^, who belong to groups Child–Turcotte–Pugh (CTP) A or B, using screening tools such as Subjective Global Assessment (SGA) or the Royal Free Hospital Nutritional Prioritizing Tool (RFH-NPT), while patients who belong to group CTP C or patients with a BMI < 18.5 kg/m^2^ are automatically labeled as at a high risk of malnutrition [8,10] (Table 2, Scheme 1). High-risk patients further undergo detailed nutritional and muscle mass assessment, accompanied by a nutritional consultation on dietary intake [8].

The RFH-NPT showed good performance in the detection of sarcopenia, which was better when compared to other models or anthropometric methods (AUROC 0.86, *p* < 0.0001) [17]. Additionally, RFH-NPT proved to correlate well with CTP and Model for End-Stage Liver Disease (MELD) scores, as well as complications of cirrhosis such as ascites, hepatorenal syndrome (HRS), and hepatic encephalopathy [18]. It serves as an independent prognostic factor for clinical deterioration and overall survival, with an associated hazard ratio (HR) of 1.59–3.58 [18,19].

A quick and simple questionnaire, the SARC-F score is frequently used to exclude sarcopenia in patients with liver cirrhosis, due to its high sensitivity (80%), whereas its specificity is rather low (50%) [20,21,22] (Table 2).

## 4. Assessment of Muscle Mass

As per the definition itself, the assessment of sarcopenia includes both the measurement of muscle mass and the estimation of muscle function. The aforementioned includes the assessment of muscle strength and physical performance [23,24].

Muscle mass can be evaluated through various validated methods such as multislice computed tomography (MSCT), abdominal magnetic resonance imaging (MRI), dual X-ray absorptiometry (DXA), bioelectrical impedance analysis (BIA), or muscle ultrasound [23] (Table 3). 

Muscle mass may be quantified as total skeletal muscle mass (SMM), as appendicular skeletal muscle mass (ASM), or as the area of muscle tissue on a given cross-section [21]. Since the amount of skeletal muscle tissue usually correlates with body size, indices, in which skeletal mass is divided by the square of body height, body weight, or BMI, have been introduced [25].

### 4.1. Multislice Computed Tomography: L3 SMI

Clinical guidelines have established the evaluation of skeletal muscle index (SMI) at the third lumbar vertebral level using abdominal MSCT (L3-SMI) as the reference standard for sarcopenia diagnosis [21,23,26]. Research has demonstrated that evaluating both muscle mass and adipose tissue obtained from a single cross-section correlates very well with the overall distribution of fat and skeletal muscle [21,27,28].

**Figure 1:**
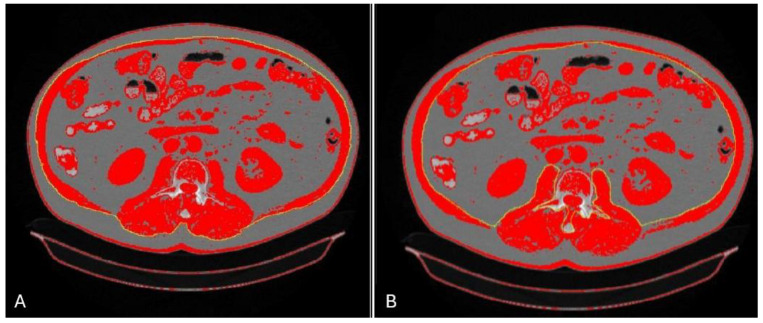
L3-SMI in the assessment of sarcopenia using ImageJ software version 1.48: outer (**A**) and inner (**B**) outline of muscles at the third lumbar section. L3-SMI: skeletal muscle index at the level of the third lumbar vertebra.

After performing abdominal MSCT, a transverse section is extracted at the level of the third lumbar vertebra, which is then implemented in dedicated software, and the desired muscle tissue is outlined (corresponding to the Hounsfield units (HU) of −29 to 150 HU). Muscle tissue includes the psoas, quadratus lumborum, erector spinae, transversus abdominis, internal and external oblique muscles, and rectus abdominis. This area (cm^2^) is then divided by the square of the body height (m^2^) to provide the SMI (Figure 1). 

For patients with liver cirrhosis, Prada’s L3-SMI cut-off values proposed in 2008 were used initially. They emerged from a study conducted in Canada on 250 patients with malignant tumors of the respiratory and gastrointestinal (GI) tract [29]. The proposed cut-off values (<52.4 cm^2^/m^2^ for men and <38.5 cm^2^/m^2^ for women) were established based on thresholds that demonstrated a significant correlation between reduced muscle mass and increased mortality risk using the optimal stratification analysis [29].

In 2012, Montano-Loza and colleagues carried out a study involving 112 patients with liver cirrhosis who were listed for liver transplantation [30]. They defined sarcopenia by Prada’s cut-off values. Using the multivariate analysis, the authors demonstrated that CTP score (HR = 1.85, *p* = 0.04), MELD score (HR = 1.08, *p* < 0.001), and sarcopenia (HR = 2.21, *p* = 0.008) independently influence mortality, whereas a weak correlation of sarcopenia with these indices was observed. Consequently, they concluded that CTP and MELD scores should include sarcopenia, in order to better assess outcomes of patients with liver cirrhosis [30].

In 2013., Martin et al. proposed new cut-off values (<43 cm^2^/m^2^ for men with BMI < 25 kg/m^2^, <53 cm^2^/m^2^ for men with BMI ≥ 25 kg/m^2^; <41 cm^2^/m^2^ for women) based on a study conducted in Canada on a cohort of 1473 patients with respiratory and GI malignancies [31,32]. Following their own conclusion based on the study from 2012, regarding the need to implement sarcopenia in scoring systems, Montano-Loza introduced the term MELD-sarcopenia score (MELD-sarcopenia score = MELD + 10.35 × sarcopenia (1: sarcopenia present; 0: sarcopenia absent) [33]. The development of this scoring system was based on Martin’s threshold values for identifying sarcopenia [31,33]. The authors demonstrated the superiority of the MELD-sarcopenia score over the MELD score in terms of outcome prediction, especially in individuals with a lower MELD score, individuals presenting with refractory ascites, or those with a prior episode of variceal hemorrhage [31].

Carey et al. first proposed cut-off values in 2017, based on a study involving 396 patients with liver cirrhosis from five transplant centers in North America. The proposed cut-off values (SMI < 50 cm^2^/m^2^ for men and <39 cm^2^/m^2^ for women) were endorsed by the EASL and the American Association for the Study of Liver Disease (AASLD). However, van Vugt et al. reported no significant difference in survival outcomes between patients with and without sarcopenia when applying Carey’s proposed cut-off values [32,34].

Resorlu et al. investigated structural changes in the paravertebral muscles of patients with ankylosing spondylitis. Measurements revealed muscle atrophy and increased fatty infiltration (except at the L3–L4 level) correlating with disease duration. These findings suggest that chronic inflammation, fibrosis, reduced mobility, and altered posture contribute to muscle degeneration, which can be mirrored in the muscle degeneration processes seen in liver cirrhosis [35].

### 4.2. Multislice Computed Tomography: Psoas and Paraspinal Muscles

An alternative approach for evaluating muscle mass via abdominal MSCT involves measuring the psoas muscle area (PMA), which is determined by outlining and calculating the cross-sectional area of the psoas muscle at the L3 vertebral level. Psoas muscle index is then obtained after dividing the PMA by the square of an individual’s height (PMA/height^2^) [36].

A simpler and faster modality is to determine the axial psoas muscle thickness (APMT) and transversal psoas muscle thickness (TPMT). APMT corresponds to the largest diameter on the axial section (mm), and TPMT corresponds to the diameter perpendicular to the axial (mm). The corresponding indices can be derived from these values when normalized to height (axial psoas thickness index (APTI) and transverse psoas thickness index (TPTI)).

The psoas muscle was chosen as a simpler diagnostic modality compared to the L3-SMI since it is easily identified on MSCT sections but also represents a deep muscle that, in contrast with parietal muscles, remains largely unaffected by abdominal distension caused by ascites [37]. Huguet et al. examined the association between two psoas indices and survival in a cohort of 173 patients with liver cirrhosis on the transplant list and demonstrated a statistically significant association between low TPTI and increased risk of death (HR 0.87, 95% CI 0.76–0.99, *p* = 0.034) [38]. Durand et al. reported a 15% rise in mortality for each unit decrease in TPTI [37]. Ebadi et al. demonstrated poor performance of PMI in survival prediction in a cohort of 353 patients from four North American transplant centers, although they found a moderately strong correlation with the L3-SMI (r > 0.7, *p* < 0.001). On the other hand, Golse et al. demonstrated better survival prediction of PMA compared to the L3-SMI on a cohort of 256 patients (AUC 0.753 vs. 0.707) and proposed cut-off values for PMA: 1561 mm^2^ (Se = 94%, Sp = 57%) for men and 1464 mm^2^ (Se = 52%, Sp = 91%) for women [39].

Paternostro et al. validated the TPMI and paraspinal muscle index (PSMI) on a cohort of 109 patients with liver cirrhosis using the L3-SMI as a reference method [40]. The paraspinal muscle index was defined as the bilateral total paraspinal muscle area at the L3 level (psoas major and minor, quadratus lumborum, transversospinal muscles, and erector spinae) [40]. The authors proposed cut-off values for the TPMI: <10.7 for men and <7.8 mm/m for women, and for PSMI: <26.3 for men and <20.8 cm^2^/m^2^ for women. Both indices showed a good performance (AUCs > 0.7, *p* < 0.001). In the multivariate analysis, only TPMI proved to be an independent predictor of survival (HR = 2.82, 95% CI 1.20–6.67, *p* = 0.018), in contrast with the L3-SMI and PSMI [40].

Wang et al. employed deep convolutional neural networks with CT imaging to automate psoas major muscle mass assessment, using manual segmentation as the reference. This method showed strong agreement with manual measurements and improved both efficiency and consistency, offering a promising prognostic tool for clinical use [41].

While assessing psoas muscle thickness or area offers a quicker and more straightforward alternative to L3-SMI, clinical studies have reported inconsistent performance outcomes. Additionally, EASL guidelines do not recommend the use of the psoas muscle in the diagnosis of sarcopenia because of its constant changes due to the excessive influence of metabolic disorders that occur in liver cirrhosis [8].

### 4.3. Magnetic Resonance Imaging

Magnetic resonance imaging (MRI) allows for quantitative as well as qualitative (muscle disruption, edema, fatty infiltration, and fibrosis) assessment of muscle tissue [42].

As a comment on van Vugt’s study on the predictive value of L3-SMI on outcomes of patients with decompensated liver cirrhosis, Tandon et al. published a short retrospective study on 61 patients with the aim of comparing L3-SMI obtained by means of MSCT and MRI. The authors highlighted the disadvantages of MSCT in terms of patient exposure to contrast and radiation, as well as the need for the implementation of MRI in the diagnosis of sarcopenia, taking into account the large number of patients who will undergo this method during the diagnosis of focal liver lesions [43]. The authors demonstrated an excellent intraclass correlation coefficient (ICC) of 0.98 (95% CI = 0.96–0.99, *p* = 0.001) between the two methods, with high intra- and inter-observer reliability [43]. All of the above supports the possibility of using both radiological methods to calculate L3-SMI with equal reliability.

Since the L3 vertebral level typically falls outside the field of view in standard liver MRI protocols, Xu et al. investigated the association between the L3-SMI and the SMI between Th12, L1, and L2 levels and demonstrated a correlation of 0.977 with good performance (AUROC 0.81–0.95, Se 88–97%, and Sp 71–93%). Since the first lumbar vertebra is usually included in standard scans, the authors concluded that the L1-SMI is the most clinically applicable variant and proposed cut-off values of 43.24 cm^2^/m^2^ for men and 33.73 cm^2^/m^2^ for women [44]. Furthermore, abdominal MRI can also be used to calculate the psoas index. Beer L. et al. retrospectively investigated the predictive value of the TPMI in a cohort of 265 patients with advanced fibrosis, cACLD, or decompensated liver cirrhosis. The authors found that sarcopenia was a risk factor for mortality in patients with cACLD (HR 3.13, 95% CI: 1.33–7.41, *p* = 0.009) and decompensated cirrhosis (HR 2.45, 95% CI: 1.32–4.57) in the univariate analysis but persisted as an independent risk factor the multivariate analysis only in patients with cACLD (aHR: 2.76, 95% CI: 1.02–7.42, *p* = 0.045) [45].

A recent study shifted the paradigm in the diagnosis of sarcopenia. The authors investigated thigh muscle composition and its relationship with muscle function using the AMRA^®^ MAsS (muscle assessment score). Among 18 participants, 41% had impaired muscle composition in the form of high fat infiltration, and this group showed significantly worse physical performance evaluated with a 6 min walk test (397 m versus 470 m in the control group, *p* < 0.01). More than 40% of patients with impaired muscle composition had undergone large volume paracentesis (LVP) in the last 3 months, compared to 0% in the control group (*p* < 0.01). The presumed causes included severe portal hypertension, protein loss in ascitic fluid, and intestinal edema that reduces the absorption of nutrients. The AMRA^®^ MAsS Scan may help lower overall healthcare costs by reducing scan duration to under 15 min and eliminating the need for manual body composition analysis by radiologists [46].

MRI can serve as a method complementary to MSCT in the assessment of muscle mass, usually in patients who undergo MR under suspicion of hepatocellular cancer (HCC). Nevertheless, utilizing MRI exclusively for the diagnosis and monitoring of sarcopenia would impose an unsustainable financial burden on healthcare systems.

### 4.4. Bioelectrical Impedance Analysis

**Figure 2:**
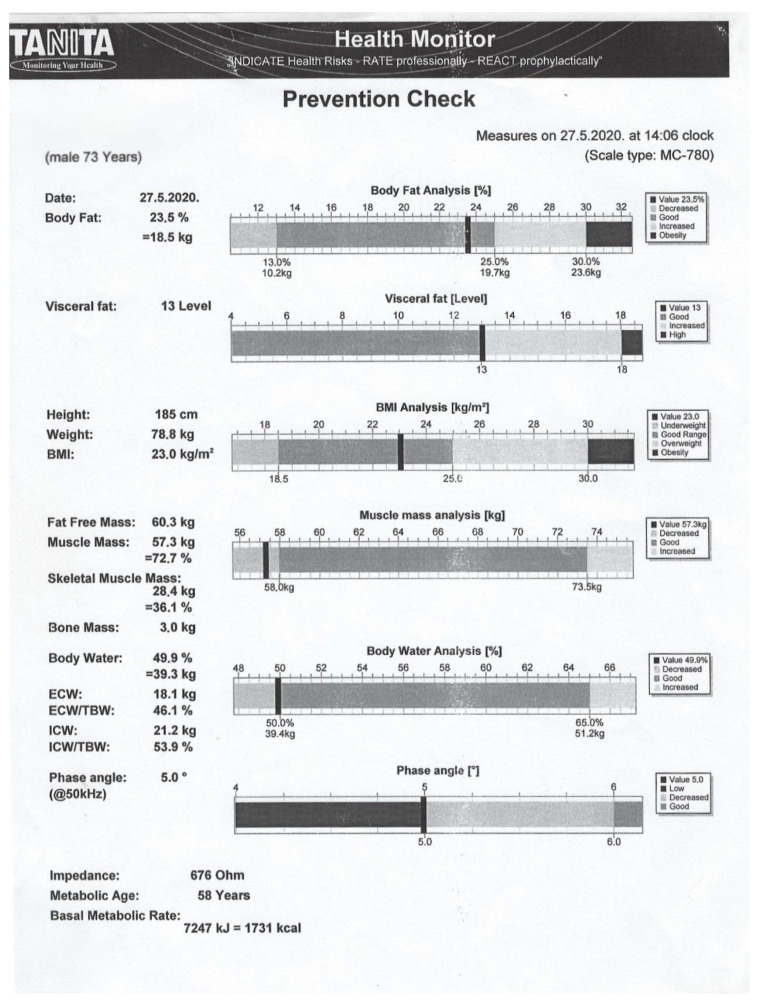
BIA parameters in the assessment of sarcopenia include SMI derived from SMM (SMM/height^2^) and the PA. This patient (SMI 8.29 kg/m^2^, PA 5.0°) would be classified as sarcopenic.

Bioelectrical impedance analysis (BIA) is a method that calculates body composition using a unique technique of passing a low-voltage alternating current through the body, while the device measures resistance and reactance, thereby allowing the calculation of body water, skeletal muscle mass, fat-free mass, and phase angle [47]. BIA consists of eight tactile electrodes embedded in a stainless-steel base and coated handles (two electrodes for each leg/arm). The most important parameters obtained by means of BIA include skeletal muscle mass (SMM), skeletal muscle index (SMI), and phase angle (PA) (Figure 2). 

When determining SMM, BIA software uses one of the existing models according to Kyle, Jassen, Ergo, or Scafoglieri [48]. The most commonly used equation for Caucasian subjects is Jansen’s: SMM (kg) = [(Ht^2^/R × 0.401) + (sex × 3.825) + (age × −0.071)] + 5.102, where Ht is height in centimeters, R is resistance in ohms, men = 1, and women = 0 [49]. Skeletal muscle index (SMI) is then calculated as the ratio of SMM to the square of height in centimeters. One of the main controversies of BIA is the influence of ascites and leg edema on the calculation of body composition. The Japanese Society of Hepatology concluded that ascites has minimal influence on sarcopenia assessment. This was supported by a study involving 149 cirrhotic patients, which demonstrated a strong correlation (R = 0.72) between BIA-derived SMI and L3-SMI, irrespective of ascites presence [50]. In a study involving 106 Caucasian patients with liver cirrhosis, a moderate correlation (r = 0.509) was observed between the assessment methods, with a stronger correlation (r = 0.614) noted in those without ascites or with only mild ascites or peripheral edema [51]. The authors also proposed a cut-off value for BIA-SMI of <11.1 kg/m^2^ (AUROC 0.737, 95% CI (0.643–0.831); *p* < 0.001) [51].

Phase angle measures the relationship between resistance and reactance via the formula: PhA = arctangent (reactance (Xc)/resistance (R)) × (180°/π)). Resistance is a measure of the amount of electric current that a substance will stop, and reactance is the ability of a substance to slow down the electric current and store a part of that charge, or, in other words, to act as a capacitor. In the human body, cell membranes act as capacitors. The slowed current lags behind the voltage, which results in a phase shift. Since alternating current has a sine wave, this shift is measured in degrees (°) and is described as the phase angle (α) [52]. The phase shift therefore measures the ability of a cell to act as a capacitor. A greater ability of the cell membrane to store electrical energy reflects its proper function and results in a higher PA. In the case of defective integrity of the cell membrane or an imbalance in body fluids (e.g., in cases of malnutrition or disease), the membrane loses its ability to store electrons and can no longer function as an efficient capacitor; therefore, PA decreases. Good correlation of PA with the L3-SMI has been described in the literature (r = 0.57–0.58) [51,53]. Several studies have also proposed cut-off values for PA ranging from ≤4.9° to 5.6° in cirrhotic patients, among which two studies suggested the same cut-off value of ≤5.05° [51,53,54,55,56,57].

### 4.5. Dual X-Ray Absorptiometry

Dual-energy X-ray absorptiometry (DXA) utilizes low-dose X-rays at two distinct energy levels—typically 40 and 70 kV—to generate whole-body imaging for body composition analysis. These radiations are absorbed or scattered differently depending on the energy level and the structure of the tissue they encounter. For example, soft tissue causes a lower attenuation of the radiation beam compared to the bone. DXA calculates the R value, which is the ratio of X-ray attenuation at two different energy levels. This ratio is unique to each tissue type and enables the differentiation between bone, fat, and lean mass [58].

DXA thus measures the proportion of lean mass (LM), fat mass (FM), and bone mineral content (BMC). LM of the upper and lower limbs is also called appendicular lean mass (ALM), which consists of four compartments: skeletal muscle tissue of the limbs, intramuscular fat, connective tissue, and skin with the subcutaneous fat. It is considered that the skeletal muscle tissue of the limbs makes up 85% of ALM, and thus, DXA indirectly quantifies muscle mass. However, with aging, the proportion of skeletal muscle tissue decreases, and the proportion of connective and fatty tissue increases, so DXA can overestimate the proportion of skeletal muscle tissue [59]. When ALM or ASM (appendicular skeletal mass) is quantified according to height, we obtain the ALMI (appendicular lean mass index) or the ASMI (eng. appendicular skeletal mass index). The European Working Group on Sarcopenia in Older People (EWGSOP) suggested the threshold value for DXA-ASMI of <7.26 kg/m^2^ [60].

FFMI (fat-free mass index) and ASMI obtained by means of DXA showed a weak correlation (FFMI ro = 0.44 for men and 0.54 for women; ASMI ro = 0.37 for men, 0.56 for women) with L3-SMM in a study conducted on 59 patients with decompensated liver cirrhosis. However, FFMI proved to be an independent predictor of patient survival before and after orthotopic liver transplantation (OLT) (HR = 1.12; *p* < 0.001) [61]. Sinclair et al. demonstrated a better negative predictive value of one-year survival for ULLM (upper limb lean mass) compared to ALM (HR 0.38 vs. HR 0.78) in a cohort of 420 men with decompensated liver cirrhosis [62]. Eriksen et al. obtained similar results in a cohort of 315 patients with liver cirrhosis and 315 healthy controls, since they found a stronger negative predictive value of the ULLMI (upper limb lean mass index) vs. the ASMI (HR 0.37 vs. HR 0.74) [63]. A group of Brazilian experts demonstrated that ULLM and ULLMI, unlike ALM and ALMI, are significantly associated with mortality (*p* = 0.007 and 0.001 vs. *p* = 0.330 and 0.377) and proposed cut-off values for the ULLMI (2.014 kg/m^2^ for men and 1.506 kg/m^2^ for women), with a moderately good performance (AUROC 0.62 and 0.68) [64].

The experts consider that the ULLM/ULLMI have greater clinical potential in predicting survival due to the possible influence of leg edema on the calculation of ALM/ALMI [63]. On the other hand, Belarmino et al. demonstrated that ascites and leg edema do not influence ASMI in a cohort of 144 men with liver cirrhosis. They also confirmed the predictive value of ASMI on patient mortality, especially in combination with HGS (hand-grip strength) (HR 1.03 (95% CI 1.00–1.05), *p* = 0.019) [65]. The authors proposed new cut-off values for ASMI (<7 kg/m^2^) and HGS (<25 kg), which are of clinical importance since they relate specifically to patients with liver cirrhosis [60,65]. The aforementioned studies predominantly demonstrate the greater power of ULMMI over other DXA parameters. Establishing specific cut-off values for the ULLMI is essential for accurately identifying sarcopenia, using L3-SMI as the reference standard.

### 4.6. Ultrasound Assessment of Skeletal Muscle Tissues

In addition to determining muscle mass, ultrasound assessment of skeletal muscle tissue also provides information on the quality of muscle tissue, i.e., the proportion of intramuscular fat and connective tissue. The usually evaluated muscles are the penniform muscles, such as the quadriceps femoris [21]. In addition, some experts recommend contrast-enhanced ultrasound of vascular structures and elastography of the quadriceps femoris [66].

**Figure 3:**
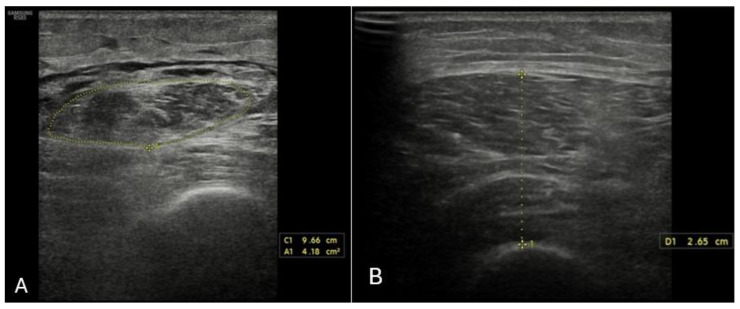
Ultrasound assessment of the quadriceps femoris muscle indicates (**A**) the absence of sarcopenia in a female patient with liver cirrhosis (CSA 4.18 cm^2^) and (**B**) the presence of sarcopenia in another female with liver cirrhosis (quadriceps thickness/height 1.56 cm/m).

**Figure 4:**
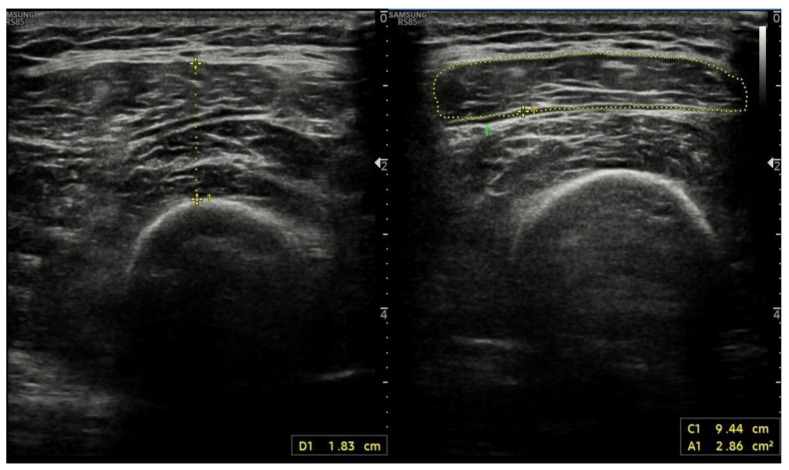
Ultrasound assessment of the quadriceps femoris muscle indicates severe sarcopenia in this male patient with alcoholic liver cirrhosis (quadriceps femoris thickness/height 1.05 cm/m and CSA 2.86 cm^2^).

Ultrasound assessment of muscle mass is widely available, fast, and non-invasive, does not expose the examiner or the subject to radiation, and allows for the assessment of intramuscular fat. However, it requires specific knowledge and experience of the examiner and the cooperation of the subject. The greatest disadvantage of this method is the undefined threshold values that would allow its use in everyday practice. Certain muscle groups, such as the psoas major or abdominal muscles, are not considered adequate for analysis due to the possible influence of ascites and excessive fat tissue. Limb muscles are therefore usually analyzed using a linear (5–12 MHz) probe, and the following parameters are determined: muscle thickness, bundle length, fiber angle, echo intensity, and the cross-sectional area (CSA) (Figure 3 and Figure 4) [67,68].

Tandon et al. proposed a model for the detection of sarcopenia based on BMI and ultrasound assessment of thigh muscle thickness, which was based on a study of 159 patients with liver cirrhosis, where L3-SMI was used as the reference method [69]. The model showed excellent diagnostic performance, with an AUROC of 0.78 in men and 0.89 in women. Regarding the assessment of thigh muscle thickness, two points were used: at one-third and one-half of the distance between the top of the patella and the iliac crest. In addition, according to the latest standards, muscle thickness was measured in two ways: with probe pressure to maximum muscle compression and without compression. The mean values were then corrected according to the square of the height in order to obtain the appropriate indices [69].

A recent study suggested rectus femoris cross-sectional area cut-off values of ≤3.48 cm^2^ for men and ≤2.97 cm^2^ for women to identify hospitalized patients at risk of malnutrition [70]. This multicenter study, involving 991 patients, found that the cross-sectional area (CSA) of the rectus femoris muscle significantly correlated with body cell mass measured by means of BIA and with handgrip strength [70].

There are no specifically proposed cut-off values for the CSA in patients with liver cirrhosis. However, a recent study conducted in Germany on a group of 63 patients with liver cirrhosis proposed a cut-off value of quadriceps femoris thickness standardized per height of ≤1.83 cm/m [67]. Patients with sarcopenia showed significantly higher rates of acute decompensation (38.9% vs. 3.7%, *p* = 0.001) and one-year mortality (27.8% vs. 3.7%, *p* = 0.013) [67]. Rectus abdominis thickness is also considered a potential predictor of clinical outcomes (HR = 0.701; 95% CI 0.533–0.922; *p* = 0.011), and the psoas index a predictor of hospitalization (HR = 0.881, 95% CI: 0.836–0.929, *p* < 0.0001) and mortality (HR = 0.930, 95% CI: 0.876–0.987, *p* = 0.017) in patients with decompensated liver cirrhosis [71,72]. However, there are currently no standardized rules regarding the choice of muscle, measurement point, measured parameters, or clinically tested cut-off values for the detection of sarcopenia in patients with liver cirrhosis. This imposes the need for larger clinical trials, given the promising diagnostic potential of this method.

### 4.7. Anthropometric Methods

Anthropometric methods include measurements of BMI, mid-arm circumference (MAC), waist circumference (WC), calf circumference (CC), and triceps skinfold thickness (TSF), as well as the calculation of MAMC (Mid Arm Muscle Circumference): MAC- (3.1415xTSF) [42,73].

MAC is the circumference of the upper arm between the tip of the olecranon and the acromion with the arm bent at 90°. Triceps skinfold thickness (TSF) is measured at the midpoint between the acromion and the olecranon with the arm relaxed at the side, using calipers to assess the thickness of the subcutaneous fat layer. For both methods, the mean of the three measurements is taken as the final value. The advantage of these methods in patients with liver cirrhosis is the absence of volume load influence, fast performance, low cost, and excellent interobserver reliability when performed by trained personnel (correlation 0.8 for TSF and 0.9 for MAMC) [8,74].

**Table 3:** Characteristics of methods used in sarcopenia assessment of patients with liver cirrhosis [75].

Method	Advantages	Disadvantages	Cut-Off
MSCT (L3-SMI)	Accuracy Reproducibility High resolution Different tissues on an anatomical level	Long learning curve Requires experience of the examiner Time consuming Expensive Radiation exposure Contraindicated in pregnancy	L3-SMI: <50 cm^2^/m^2^ (M) and <39 cm^2^/m^2^ (W) [32] PMA: 1561 mm^2^ (M) and 1464 mm^2^ (W) [39] TPMI: <10.7 mm/m (M) and <7.8 mm/m (W) [40] PSMI: <26.3 cm^2^/m^2^ (M) and <20.8 cm^2^/m^2^ (W) [40]
MRI	Accuracy Reproducibility High resolution Different tissues on an anatomical level	Long learning curve Requires experience of the examiner Time consuming Expensive Not widely available	L1-SMI: 43.24 cm^2^/m^2^ (M) and 33.73 cm^2^/m^2^ (W) [44]
DXA	Fast and simple Minimal radiation exposure Low cost	Requires experience of the examiner Contraindicated in pregnancy Does not differentiate between subcutaneous, visceral, and intramuscular fat	ULLMI: 2.014 kg/m^2^ (M) and 1.506 kg/m^2^ (W) [64] ASMI: <7 kg/m^2^ [65]
BIA	Fast and very simple No radiation exposure Low cost Widely available	Impact of ascites and edema	BIA-SMI: <11.1 kg/m^2^ [51] PA: ≤4.9–5.6° [51,53,54,55,56,57]
US	US used routinely by hepatologists No radiation exposure Low cost Widely available		QFTI: ≤1.83 cm/m [67]
Anthropometric methods	Fast and simple Low cost	High interobserver variability Low reproducibility	

MSCT: multislice computed tomography, MRI: magnetic resonance imaging; DXA: dual X-rax absorptiometry; BIA: bioelectrical impedance analysis; US: ultrasound; L3-SMI: skeletal muscle index at the level of third lumbar vertebra; PMA: psoas muscle area; TPMI: transversal psoas muscle index; PSMI: paraspinal muscle index; L1-SMI: skeletal muscle index at the level of first lumbar vertebra; ASMI: appendicular skeletal mass index; ULLMI: upper limb lean mass index; PA: phase angle; BIA-SMI: bioelectrical impedance analysis skeletal muscle index; QFTI: quadriceps femoris thickness index.

Saueressig C et al. proposed threshold values for MAMC based on a study conducted on a cohort of 1075 patients with liver cirrhosis: ≤21.5 cm for moderate and ≤24.2 cm for severe malnutrition in women; ≤20.9 cm for moderate and ≤22.9 cm for severe malnutrition in men [76].

Regarding the comparison of MAMC with the reference method, a study conducted on 59 patients listed for OLT found a weak correlation between methods (0.48 for men; 0.18 for women) [77]. Similar results were presented by Nishikawa H. et al., who investigated several anthropometric parameters (MAC, MAMC, CC (calf circumference), MAMA, TSF, WC (waist circumference), and BMI) on a group of patients with liver cirrhosis (103 men and 87 women) [78]. However, CC showed excellent correlation with L3-SMI (r = 0.79, *p* < 0.0001 in men, r = 0.83, *p* < 0.0001 in women), as well as the best diagnostic performance (AUROC 0.88 in men and 0.86 in women) [78]. Despite the weak correlation, MAMC and TSF showed satisfactory diagnostic performance [78].

A similar study was conducted in Romania on a cohort of 156 patients with liver cirrhosis. Sarcopenia was defined by low L3-SMI (according to Carey), accompanied by decreased muscle strength (HGS) [17]. The authors investigated the correlation and performance of a number of anthropometric methods, whereby RFH-NPT, MAC, and MAMC showed the best diagnostic accuracy (AUROC 0.86, 0.81, and 0.79, respectively). TSF, on the other hand, showed poor performance (AUROC 0.63) [17].

Almost 30 years ago, Merli et al. demonstrated the association between MAMC and survival in a cohort of 1492 patients with liver cirrhosis [79]. In 2001, Alberino and colleagues confirmed these results on a cohort of 212 hospitalized patients with liver cirrhosis. Using TSF and MAMC measurements, researchers demonstrated that patients with moderate-to-severe malnutrition had significantly lower survival rates at 6, 12, and 24 months compared to those with adequate or excessive nutritional status (*p* < 0.05) [61]. Saueressig C et al. demonstrated that a marked decrease in MAMC is an independent predictor of one-year mortality (HR: 1.71, 95% CI: 1.24–2.35, *p* < 0.001), while each 1 cm increase in MAMC was associated with an 11% reduction in mortality risk (HR: 0.89, 95% CI: 0.85–0.94, *p* < 0.001) [76]. A decreased MAMC is also a significant negative prognostic factor for survival following orthotopic liver transplantation (OLT) [77].

Although fast and easy to perform, anthropometric methods are recommended as the last option for the assessment of muscle mass according to EASL, primarily due to wide interobserver variability and their inability to make a distinction between muscle mass and fat tissue.

## 5. Assessment of Muscle Function

Assessment of muscle function involves testing muscle strength and physical performance.

### 5.1. Muscle Strength

Handgrip strength (HGS) and the chair rise test are commonly employed to evaluate muscle strength. HGS, in particular, is a straightforward and cost-effective method that only requires a calibrated dynamometer. The test is performed by having the subject hold the dynamometer in their hand lying on their side, with the elbow at 90°, and squeeze it as hard as possible for about 3 s. The average of the measurements is taken from three attempts and then compared with the normal ranges for age and gender. The proposed cut-off value for the elderly population according to EWGSOP is <30 kg, while Belarmino et al. proposed a cut-off value of <25 kg specific to patients with liver cirrhosis [65]. The HGS proved to be a good predictor of the hospitalization duration, functional limitations, quality of life, and outcomes [21].

Tapper et al. found a good correlation between HGS and the muscle mass area obtained by means of MSCT (r = 0.64, *p* < 0.001) in a cohort of 106 patients with compensated liver cirrhosis [80], as did Luengpradidgun L. et al., who, in addition to a strong correlation with L3-SMI (r = 0.81, *p* < 0.001), also demonstrated excellent diagnostic performance of HGS (Se 88.2%, Sp 100%, NPV 100%, PPV 98.7%) [81]. Certain authors demonstrated satisfactory performance of HGS (AUROC 0.73) despite a weaker correlation with the L3-SMI (0.31, *p* = 0.001) and proposed a cut-off value of ≤31 kg (Se 90.2%, Sp 25.4%, PPV 30.3%, NPV 87.9%) for men with decompensated liver cirrhosis. HGS serves as an independent predictor of survival in patients with liver cirrhosis, with each unit increase in HGS associated with reduced mortality risk in both men (HR 0.96; 95% CI 0.94–0.99, *p* < 0.01) and women (HR 0.91; 95% CI 0.84–0.99, *p* = 0.02) [82].

The chair rise test can be used as an alternative method to assess the strength of the leg muscles or the quadriceps group. This test evaluates lower limb strength by timing how long it takes a person to stand from a seated position five times without using their hands, or alternatively, by counting the number of sit-to-stand repetitions completed within 30 s [21]. There are currently no studies that have evaluated this method in the assessment of sarcopenia in patients with liver cirrhosis.

### 5.2. Physical Performance

Physical performance refers to the objective assessment of overall body function, primarily focusing on mobility and functional capacity [21]. In addition to evaluating muscle function, this concept also encompasses the evaluation of both central and peripheral nervous system function, which are important in the execution of movements and maintenance of balance. A method that is widely accepted in testing physical performance is the gait speed test. This safe, simple, reliable, and rapid test involves the time required for an individual to walk a distance of 4 m at a normal pace. A speed cut-off value of ≤0.8 m/s is recommended by the EWGSOP2 as an indicator of severe sarcopenia [21].

Other alternative tests include the TUG test (timed up and go) and the SPPB test (short physical performance battery). The TUG test and the SPPB are designed as combinations of several tasks, e.g., the SPPB includes walking speed, a balance test, and a test of getting up from a chair. The total possible score is 12, with a score below 8 suggesting the presence of sarcopenia. The TUG test involves standing up from a chair, walking 3 m, turning around, returning to the chair, and sitting down [83,84]. Soto et al. tested the physical performance of 126 patients with liver cirrhosis. As many as 26.9% of patients had reduced walking speed (≤0.8 m/s) and higher mortality compared to the group with normal walking speed (80% vs. 40%, *p* < 0.001) [85]. In multivariate analysis, reduced gait speed and weakness were detected as negative prognostic factors (HR = 3.27, 95% CI 1.74–6.14, *p* < 0.001 and HR = 4.24, 95%CI 1.89–9.51, *p* < 0.001) [85].

## 6. Discussion

Sarcopenia plays a critical role in deteriorating the clinical course, compromising quality of life, and negatively affecting outcomes in patients with liver cirrhosis. Two large meta-analyses involving 6965 and 4037 cirrhotic patients reported high sarcopenia prevalence (37.5% and 48%), both showing a strong, independent association with increased mortality risk (HR 2.30 and HR 1.72, *p* < 0.001) [6,86]. Tantai et al. found that the mortality rate increased with the severity or duration of sarcopenia. Cumulative 1-year, 3-year, and 5-year survival rates were 16.8%, 17.7%, and 28.9% lower in individuals with sarcopenia, and each 1 cm^2^/m^2^ increase in L3-SMI reduced the risk of mortality by 3% [6]. In a cohort of 307 cirrhotic patients with serial CT scans, sarcopenia status was categorized over a 6-month interval. Persistent and new-onset sarcopenia were significantly associated with reduced survival compared to non-sarcopenic groups [87].

Kim et al. demonstrated that changes in muscle mass over a one-year period were significantly linked to the development of cirrhosis-related complications, independent of CTP and MELD-Na scores. The ΔSMI/year model—calculated as ((SMI at one year − SMI at baseline)/SMI at baseline × 100 (%))—proved effective in predicting complications within six months, with a cut-off value of −2.62, yielding an AUROC of 0.817, sensitivity of 83.9%, and specificity of 74.5% [88]. A single-center study of 215 cirrhotic patients on the LT waitlist developed the Sarcopenia HIBA score to predict sarcopenia using clinical variables: male sex, BMI, Child–Pugh score, and creatinine/Cystatin C ratio. The score demonstrated strong diagnostic performance (AUC 0.862) and was independently associated with waitlist mortality, even after MELD-Na adjustment. The Sarcopenia HIBA score offers a practical tool for identifying high-risk patients when CT assessment is unavailable [89].

A 2016 meta-analysis involving 3803 patients across 19 cohorts found that sarcopenia significantly increases post-liver transplantation (LT) mortality, with a pooled hazard ratio (HR) of 1.84. Sarcopenia is associated with heightened susceptibility to infections, extended ICU stays, greater need for mechanical ventilation, and increased transfusion requirements [90,91,92]. A systematic review of 12,276 records identified 34 relevant studies assessing the impact of frailty and sarcopenia on liver transplantation outcomes. Both conditions were associated with significantly worse post-transplant prognosis, including up to a two-fold reduction in early and a 50% reduction in late survival in severe cases. Affected patients experienced longer ICU and hospital stays, increased rates of sepsis and respiratory complications, and reduced graft survival. The findings highlight the clinical dilemma: although frailty and sarcopenia increase waitlist mortality and support prioritization for transplantation, advanced cases may warrant delisting due to poor post-transplant outcomes. Early intervention in mildly affected patients is crucial to avoid deterioration and improve prognosis [93].

In 2000, it was estimated that sarcopenia was responsible for an annual health care cost of about USD 18.5 billion in the USA, while in 2019, the average yearly expenditure for hospital treatment of patients with sarcopenia was approximately USD 40.4 billion [94,95]. Additionally, individuals with sarcopenia faced a significantly greater risk of hospitalization (OR 1.95; *p* < 0.001) and incurred an added yearly healthcare expenditure of USD 2315.70 per person compared to non-sarcopenic individuals [95]. Market assessment agencies have reported that the sarcopenia treatment market, estimated at USD 3.02 trillion in 2023, will grow to USD 4.23 trillion by 2030, with a compound annual growth rate (CAGR) of 5% [96].

Tapper et al. demonstrated that body composition analysis using clinically acquired CT scans enhances risk prediction for decompensation, outperforming the MELD score. In addition to muscle mass, the quality of both muscle and fat tissue is important for forecasting liver-related complications such as ascites and hepatic encephalopathy [97]. Although L3-SMI is considered the gold standard, the calculation process is time-consuming, and the method is not suitable for patient monitoring due to radiation exposure. The same diagnostic accuracy as MSCT is provided by MRI. In addition to quantifying muscle mass, both methods allow for qualitative assessment of muscle tissue, i.e., detection of microscopic and macroscopic changes. However, in clinical practice, both are inadequate due to poor availability and high cost, and the process of calculating muscle mass requires training and the expenditure of time. Because standardized threshold values are still lacking, MRI is primarily utilized within clinical research settings. However, recent scientific advancements are paving the way for the growing integration of artificial intelligence (AI) in analyzing body composition through both retrospective and prospective evaluations of CT and MRI data [98,99].

Unlike MSCT and MR, DXA is a much cheaper, more accessible, and faster method. However, studies have revealed unequal results among devices, which impairs defining the cut-off values [21,100]. DXA involves exposure to ionizing radiation for both the patient and the examiner, though the dose remains minimal, typically less than 10 microsieverts [58]. The main disadvantage is the potential overestimation of ALM in older age due to the increased proportion of intramuscular fat. The obvious advantage of this method includes information on bone cell mass [101]. BIA is a cheap, portable, and easily accessible method that does not emit radiation nor does it require the contrast application. Its employment can be easily repeated, which allows for patient monitoring, and is especially relevant when evaluating the impact of nutritional or physical activity-based treatment strategies. The main disadvantage is the unclear influence of ascites and limb edema on the assessment of muscle mass.

Muscle tissue ultrasound is a portable, cheap, and non-invasive method that does not expose subjects to radiation and has shown good diagnostic reliability when compared with DXA, MRI, and MSCT [60]. However, despite its many benefits, the absence of universally accepted threshold values currently limits its applicability in routine clinical settings [42,98]. Ultimately, anthropometric methods, despite their simplicity and speed, show significant interobserver variability and poor reproducibility, which is why their use is recommended only when other methods are unavailable [21].

Automated models for assessing muscle and adipose tissue using imaging, particularly CT and MRI, have shown promising results, despite the field’s novelty. These advancements rely on collaboration between radiologists, clinicians, and machine learning experts. While current sarcopenia assessment methods are simple and cost-effective, they lack precision. Integrating AI into routine imaging could enhance diagnostic accuracy. Radiomics, which transforms medical images into quantifiable data, holds particular promise. Rozynek et al. analyzed 23 studies, among which CT-based methods dominated, with many achieving high segmentation accuracy—some reporting dice similarity coefficients (DSCs) >0.95—especially at the L3 vertebral level, a key site for body composition analysis [102]. Bedrikovetski et al. conducted a systematic review and a meta-analysis of studies employing deep learning models for skeletal muscle (SM) segmentation. These models showed high segmentation accuracy for skeletal muscle (pooled DSC: 0.941), subcutaneous fat (0.967), visceral fat (0.963), and bone (0.970). Despite promising results, significant heterogeneity and publication bias were noted, particularly in muscle studies. CT-based deep learning offers strong potential for automated body composition analysis in sarcopenia, but standardized evaluation and validation are needed before clinical adoption [103].

Which of the aforementioned methods the attending physician will adopt depends on availability, financial resources, patient cooperation, previous experience, available time, and ultimately their personal discretion. If medical and time resources permit, we recommend the use of L3-SMI, recognized as the diagnostic gold standard—particularly given that most patients undergo MSCT as part of their diagnostic evaluation. In settings where this is not feasible, quicker and more accessible methods such as BIA or DXA may be suitable alternatives. Ultimately, ultrasound-based assessment may offer the most practical and familiar option for hepatologists, who routinely employ advanced ultrasound techniques. In this regard, further clinical studies are essential to establish validated cut-off values for its diagnostic use.

Considering the global healthcare impact and the significant influence on the course of disease, characteristics of simplicity, swiftness, safety, availability, reproducibility, and diagnostic accuracy are certainly the key factors to consider when choosing the proper diagnostic method for nutritional assessment.

## Figures and Tables

**Table 1:** Definition of sarcopenia by EWGSOP2, EASL, and GLIS [4,8,10].

Guidelines	Definition	Cut-Offs
EWGSOP2 (2019) [10]	Reduced muscle strength and muscle mass (or quality) Severe sarcopenia: when accompanied by reduced physical performance	Muscle strength HGS: <27 kg (M) and <16 kg (F) Stand-up test: >15 s for 5 stands Muscle mass ASM < 20 kg (M) or <15 kg (F) ASMI < 7 kg/m^2^ (M) or <5.5 kg/m^2^ (F) Physical performance Walking speed < 0.8 m/s SPPB < 8 TUG > 20 s 400 m walk test: >6 min or failure
EASL (2019) [8]	Reduced muscle mass and function	Not clearly defined.
GLIS (2024) [4]	Concurrent combination of reduced muscle mass and muscle strength	Not clearly defined

EWGSOP: European Working Group on Sarcopenia in Older People; HGS: hand-grip strength; ASM: appendicular skeletal muscle; M: male; F: female; SPPB: short physical performance battery; TUG: timed up and go; EASL: European Association for the Study of the Liver; GLIS: Global Leadership Initiative in Sarcopenia.

**Table 2:** Nutritional screening questionnaires.

Questionnaires	
SGA	SARC-F
Weight loss	Muscle strength
Dietary intake	Assistance in walking
GI symptoms	Rise from a chair
Functional status	Climbing stairs
Disease and its relation to nutritional requirements	Falls
Physical findings	

SGA: Subjective Global Assessment; GI: gastrointestinal.

## Data Availability

Not applicable.

## Abbreviations

The following abbreviations are used in this manuscript:

AASLD	American Association for the Study of Liver Disease
AI	Artificial intelligence
ALD	Alcoholic liver disease
ALM	Appendicular lean mass
ALMI	Appendicular lean mass index
APMT	Axial psoas muscle thickness
APTI	Axial psoas thickness index
ASM	Appendicular skeletal muscle mass
ASMI	Appendicular skeletal mass index
BCAA	Branched-chain amino acids
BCM	Body cell mass
BIA	Bioelectrical impedance analysis
BMC	Bone mineral content
BMI	Body mass index
CAGR	compound annual growth rate
CC	Calf circumference
CSA	Cross-sectional area
CTP	Child–Turcotte–Pugh
DAMPs	Damage-associated molecular patterns
DXA	Dual X-ray absorptiometry
EASL	European Association for the Study of the Liver
ESPEN	European Society for Clinical Nutrition and Metabolism
EWGSOP	European Working Group on Sarcopenia in Older People
FFMI	Fat-free mass index
FM	Fat mass
GI	Gastrointestinal
GLIS	Global Leadership Initiative in Sarcopenia
HCC	Hepatocellular cancer
HGF	α-hepatocyte growth factor alpha
HGS	Hand-grip strength
HRS	Hepatorenal syndrome
HU	Hounsfield units
ICC	Intraclass correlation coefficient
IGF1	Insulin growth factor 1
IκB	IkappaB kinase
L3-SMI	Skeletal muscle index at the level of the third lumbar vertebra
LH	Luteinizing hormone
LM	Lean mass
LVP	Large volume paracentesis
NF-κB	Nuclear factor kappa B
MAC	Mid-arm circumference
MAMC	Mid-arm muscle circumference
MAsS	Muscle assessment score
MELD	Model for End-Stage Liver Disease
MRI	Magnetic resonance imaging
MSCT	Multislice computed tomography
mTOR	Mammalian target of rapamycin
OLT	Orthotopic liver transplantation
PA	Phase angle
PAMPs	Pathogen-associated molecular patterns
PMA	Psoas muscle area
PMI	Psoas muscle index
PSMI	Paraspinal muscle index
RFH-NPT	Royal Free Hospital Nutritional Prioritizing Tool
SGA	Subjective Global Assessment
SMI	Skeletal muscle index
SMM	Skeletal muscle mass
SPPB	Short physical performance battery
tPMT	Transversal psoas muscle thickness
TPTI	Transverse psoas thickness index
TSF	Triceps skinfold thickness
TUG	Timed up and go
ULLMI	Upper limb lean mass index
U-P	Ubiquitin–proteasome
WC	Waist circumference

## References

[1] Tsochatzis E.A., Bosch J., Burroughs A.K. (2014). Liver cirrhosis. Lancet.

[2] GBD 2017 Cirrhosis Collaborators (2020). The global, regional, and national burden of cirrhosis by cause in 195 countries and territories, 1990-2017: A systematic analysis for the Global Burden of Disease Study 2017. Lancet Gastroenterol. Hepatol..

[3] Cederholm T., Barazzoni R., Austin P., Ballmer P., Biolo G., Bischoff S.C., Compher C., Correia I., Higashiguchi T., Holst M. (2017). ESPEN guidelines on definitions and terminology of clinical nutrition. Clin. Nutr..

[4] Kirk B., Cawthon P.M., Arai H., Ávila-Funes J.A., Barazzoni R., Bhasin S., Binder E.F., Bruyere O., Cederholm T., Chen L.-K. (2024). The Conceptual Definition of Sarcopenia: Delphi Consensus from the Global Leadership Initiative in Sarcopenia (GLIS). Age Ageing.

[5] Sayer A.A., Cooper R., Arai H., Cawthon P.M., Essomba M.-J.N., Fielding R.A., Grounds M.D., Witham M.D., Cruz-Jentoft A.J. (2024). Sarcopenia. Nat. Rev. Dis. Primers.

[6] Tantai X., Liu Y., Yeo Y.H., Praktiknjo M., Mauro E., Hamaguchi Y., Engelmann C., Zhang P., Jeong J.Y., van Vugt J.L.A. (2022). Effect of sarcopenia on survival in patients with cirrhosis: A meta-analysis. J. Hepatol..

[7] Sinclair M., Gow P.J., Grossmann M., Angus P.W. (2016). Review article: Sarcopenia in cirrhosis—Aetiology, implications and potential therapeutic interventions. Aliment. Pharmacol. Ther..

[8] European Association for the Study of the Liver (2019). EASL Clinical Practice Guidelines on nutrition in chronic liver disease. J. Hepatol..

[9] Meyer F., Bannert K., Wiese M., Esau S., Sautter L.F., Ehlers L., Aghdassi A.A., Metges C.C., Garbe L.A., Jaster R. (2020). Molecular Mechanism Contributing to Malnutrition and Sarcopenia in Patients with Liver Cirrhosis. Int. J. Mol. Sci..

[10] Fox R., Stenning K., Slee A., Macnaughtan J., Davies N. (2022). Sarcopenia in liver cirrhosis: Prevalence, pathophysiology and therapeutic strategies. Anal. Biochem..

[11] Del Cioppo S., Faccioli J., Ridola L. (2025). Hepatic Cirrhosis and Decompensation: Key Indicators for Predicting Mortality Risk. World J. Hepatol..

[12] Emanuele N.V., LaPaglia N., Benefield J., Emanuele M.A. (2001). Ethanol-induced hypogonadism is not dependent on activation of the hypothalamic-pituitary-adrenal axis. Endocr. Res..

[13] Arroyo V., Angeli P., Moreau R., Jalan R., Clària J., Trebicka J., Fernández J., Gustot T., Caraceni P., Bernardi M. (2021). The systemic inflammation hypothesis: Towards a new paradigm of acute decompensation and multiorgan failure in cirrhosis. J. Hepatol..

[14] Qiu J., Thapaliya S., Runkana A., Yang Y., Tsien C., Mohan M.L., Narayanan A., Eghtesad B., Mozdziak P.E., McDonald C. (2013). Hyperammonemia in cirrhosis induces transcriptional regulation of myostatin by an NF-κB-mediated mechanism. Proc. Natl. Acad. Sci. USA.

[15] Espina S., Sanz-Paris A., Bernal-Monterde V., Casas-Deza D., Arbonés-Mainar J.M. (2022). Role of Branched-Chain Amino Acids and Their Derivative β-Hydroxy-β-Methylbutyrate in Liver Cirrhosis. J. Clin. Med..

[16] Bischoff S.C., Bernal W., Dasarathy S., Merli M., Plank L.D., Schütz T., Plauth M. (2020). ESPEN practical guideline: Clinical nutrition in liver disease. Clin. Nutr..

[17] Topan M.M., Sporea I., Dănilă M., Popescu A., Ghiuchici A.-M., Lupușoru R., Șirli R. (2022). Comparison of Different Nutritional Assessment Tools in Detecting Malnutrition and Sarcopenia among Cirrhotic Patients. Diagnostics.

[18] Borhofen S.M., Gerner C., Lehmann J., Fimmers R., Görtzen J., Hey B., Geiser F., Strassburg C.P., Trebicka J. (2016). The Royal Free Hospital-Nutritional Prioritizing Tool Is an Independent Predictor of Deterioration of Liver Function and Survival in Cirrhosis. Dig. Dis. Sci..

[19] Tan J.Y.T., Cheah C.C.M., Wang Y.T., Chang P.E., Krishnamoorthy T.L., Tan H.K., Salazar E. (2023). Outpatient screening with the Royal Free Hospital-Nutrition Prioritizing Tool for patients with cirrhosis at risk of malnutrition. Nutrition.

[20] Singla N., Inavolu P., Kumar B.R., Macherla R., Reddy D.N. (2024). SARC-F Score: A Quick Bedside Tool to Screen Sarcopenia in Patients With Cirrhosis. J. Clin. Exp. Hepatol..

[21] Cruz-Jentoft A.J., Bahat G., Bauer J., Boirie Y., Bruyère O., Cederholm T., Cooper C., Landi F., Rolland Y., Sayer A.A. (2019). Sarcopenia: Revised European consensus on definition and diagnosis. Age Ageing.

[22] Malmstrom T.K., Miller D.K., Simonsick E.M., Ferrucci L., Morley J.E. (2016). SARC-F: A symptom score to predict persons with sarcopenia at risk for poor functional outcomes. J. Cachexia Sarcopenia Muscle.

[23] Dent E., Morley J.E., Cruz-Jentoft A.J., Arai H., Kritchevsky S.B., Guralnik J., Bauer J.M., Pahor M., Clark B.C., Cesari M. (2018). International Clinical Practice Guidelines for Sarcopenia (ICFSR): Screening, Diagnosis and Management. J. Nutr. Health Aging.

[24] Fried L.P., Tangen C.M., Walston J., Newman A.B., Hirsch C., Gottdiener J., Seeman T., Tracy R., Kop W.J., Burke G. (2001). Frailty in older adults: Evidence for a phenotype. J. Gerontol. A Biol. Sci. Med. Sci..

[25] Kim K.M., Jang H.C., Lim S. (2016). Differences among skeletal muscle mass indices derived from height-, weight-, and body mass index-adjusted models in assessing sarcopenia. Korean J. Intern. Med..

[26] Beaudart C., McCloskey E., Bruyere O., Cesari M., Rolland Y., Rizzoli R., Araujo De Carvalho I., Amuthavalli Thiyagarajan J., Bautmans I., Bertière M.-C. (2016). Sarcopenia in daily practice: Assessment and management. BMC Geriatr..

[27] Fearon K., Strasser F., Anker S.D., Bosaeus I., Bruera E., Fainsinger R.L., Jatoi A., Loprinzi C., MacDonald N., Mantovani G. (2011). Definition and classification of cancer cachexia: An international consensus. Lancet Oncol..

[28] Shen W., Punyanitya M., Wang Z., Gallagher D., St.-Onge M.-P., Albu J., Heymsfield S.B., Heshka S. (2004). Total body skeletal muscle and adipose tissue volumes: Estimation from a single abdominal cross-sectional image. J. Appl. Physiol..

[29] Prado C.M., Lieffers J.R., McCargar L.J., Reiman T., Sawyer M.B., Martin L., Baracos V.E. (2008). Prevalence and clinical implications of sarcopenic obesity in patients with solid tumours of the respiratory and gastrointestinal tracts: A population-based study. Lancet Oncol..

[30] Montano-Loza A.J., Meza-Junco J., Prado C.M., Lieffers J.R., Baracos V.E., Bain V.G., Sawyer M.B. (2012). Muscle wasting is associated with mortality in patients with cirrhosis. Clin. Gastroenterol. Hepatol..

[31] Martin L., Birdsell L., Macdonald N., Reiman T., Clandinin M.T., McCargar L.J., Murphy R., Ghosh S., Sawyer M.B., Baracos V.E. (2013). Cancer cachexia in the age of obesity: Skeletal muscle depletion is a powerful prognostic factor, independent of body mass index. J. Clin. Oncol..

[32] van Vugt J.L.A., Alferink L.J.M., Buettner S., Gaspersz M.P., Bot D., Murad S.D., Feshtali S., van Ooijen P.M.A., Polak W.G., Porte R.J. (2018). A model including sarcopenia surpasses the MELD score in predicting waiting list mortality in cirrhotic liver transplant candidates: A competing risk analysis in a national cohort. J. Hepatol..

[33] Montano-Loza A.J., Duarte-Rojo A., Meza-Junco J., Baracos V.E., Sawyer M.B., Pang J.X.Q., Beaumont C., Esfandiari N., Myers R.P. (2015). Inclusion of Sarcopenia Within MELD (MELD-Sarcopenia) and the Prediction of Mortality in Patients With Cirrhosis. Clin. Transl. Gastroenterol..

[34] Carey E.J., Lai J.C., Wang C.W., Dasarathy S., Lobach I., Montano-Loza A.J., Dunn M.A., for the Fitness, Life Enhancement, and Exercise in Liver Transplantation Consortium (2017). A multicenter study to define sarcopenia in patients with end-stage liver disease. Liver Transpl..

[35] Resorlu H., Savas Y., Aylanc N., Gokmen F. (2017). Evaluation of Paravertebral Muscle Atrophy and Fatty Degeneration in Ankylosing Spondylitis. Mod. Rheumatol..

[36] Ebadi M., Wang C.W., Lai J.C., Dasarathy S., Kappus M.R., Dunn M.A., Carey E.J., Montano-Loza A.J., From the Fitness, Life Enhancement, and Exercise in Liver Transplantation (FLEXIT) Consortium (2018). Poor performance of psoas muscle index for identification of patients with higher waitlist mortality risk in cirrhosis. J. Cachexia Sarcopenia Muscle.

[37] Durand F., Buyse S., Francoz C., Laouénan C., Bruno O., Belghiti J., Moreau R., Vilgrain V., Valla D. (2014). Prognostic value of muscle atrophy in cirrhosis using psoas muscle thickness on computed tomography. J. Hepatol..

[38] Huguet A., Latournerie M., Debry P.H., Jezequel C., Legros L., Rayar M., Boudjema K., Guyader D., Jacquet E.B., Thibault R. (2018). The psoas muscle transversal diameter predicts mortality in patients with cirrhosis on a waiting list for liver transplantation: A retrospective cohort study. Nutrition.

[39] Golse N., Bucur P.O., Ciacio O., Pittau G., Cunha A.S., Adam R., Castaing D., Antonini T., Coilly A., Samuel D. (2017). A new definition of sarcopenia in patients with cirrhosis undergoing liver transplantation. Liver Transpl..

[40] Paternostro R., Lampichler K., Bardach C., Asenbaum U., Landler C., Bauer D., Mandorfer M., Schwarzer R., Trauner M., Reiberger T. (2019). The value of different CT-based methods for diagnosing low muscle mass and predicting mortality in patients with cirrhosis. Liver Int..

[41] Wang N.C., Zhang P., Tapper E.B., Saini S., Wang S.C., Su G.L. (2020). Automated Measurements of Muscle Mass Using Deep Learning Can Predict Clinical Outcomes in Patients With Liver Disease. Am. J. Gastroenterol..

[42] Chianca V., Albano D., Messina C., Gitto S., Ruffo G., Guarino S., Del Grande F., Sconfienza L.M. (2022). Sarcopenia: Imaging assessment and clinical application. Abdom. Radiol..

[43] Tandon P., Mourtzakis M., Low G., Zenith L., Ney M., Carbonneau M., Alaboudy A., Mann S., Esfandiari N., Ma M. (2016). Comparing the Variability Between Measurements for Sarcopenia Using Magnetic Resonance Imaging and Computed Tomography Imaging. Am. J. Transplant..

[44] Xu Z., Yang D., Luo J., Xu H., Jia J., Yang Z. (2023). Diagnosis of Sarcopenia Using the L3 Skeletal Muscle Index Estimated from the L1 Skeletal Muscle Index on MR Images in Patients With Cirrhosis. J. Magn. Reson. Imaging.

[45] Beer L., Bastati N., Ba-Ssalamah A., Pötter-Lang S., Lampichler K., Bican Y., Lauber D., Hodge J., Binter T., Pomej K. (2020). MRI-defined sarcopenia predicts mortality in patients with chronic livmer disease. Liver Int..

[46] Thuluvath A.J., Forsgren M.F., Ladner D.P., Tevar A.D., Duarte-Rojo A. (2024). Utilizing a novel MRI technique to identify adverse muscle composition in end-stage liver disease: A pilot study. Ann. Hepatol..

[47] NIH Bioelectrical Impedance Analysis (BIA) Procedure [Internet]. USA: NIH. https://repository.niddk.nih.gov/media/studies/fhn_nocturnal/MOP/Chapter15_BIA.pdf.

[48] Jo M.H., Lim T.S., Jeon M.Y., Lee H.W., Kim B.K., Park J.Y., Kim D.Y., Ahn S.H., Han K.-H., Kim S.U. (2019). Predictors of Discordance in the Assessment of Skeletal Muscle Mass between Computed Tomography and Bioimpedance Analysis. J. Clin. Med..

[49] Janssen I., Heymsfield S.B., Baumgartner R.N., Ross R. (2000). Estimation of skeletal muscle mass by bioelectrical impedance analysis. J. Appl. Physiol. (1985).

[50] Nishikawa H., Shiraki M., Hiramatsu A., Moriya K., Hino K., Nishiguchi S. (2016). Japan Society of Hepatology guidelines for sarcopenia in liver disease (1st edition): Recommendation from the working group for creation of sarcopenia assessment criteria. Hepatol. Res..

[51] Bozic D., Grgurevic I., Mamic B., Capkun V., Bilandzic-Ivisic J., Ivanovic T., Bozic I., Zaja I., Podrug K., Puljiz Z. (2023). Detection of Sarcopenia in Patients with Liver Cirrhosis Using the Bioelectrical Impedance Analysis. Nutrients.

[52] Khalil S.F., Mohktar M.S., Ibrahim F. (2014). The theory and fundamentals of bioimpedance analysis in clinical status monitoring and diagnosis of diseases. Sensors.

[53] Ruiz-Margáin A., Xie J.J., Román-Calleja B.M., Pauly M., White M.G., Chapa-Ibargüengoitia M., Campos-Murguía A., González-Regueiro J.A., Macias-Rodríguez R.U., Duarte-Rojo A. (2021). Phase Angle from Bioelectrical Impedance for the Assessment of Sarcopenia in Cirrhosis with or Without Ascites. Clin. Gastroenterol. Hepatol..

[54] Ruiz-Margáin A., Macías-Rodríguez R.U., Duarte-Rojo A., Ríos-Torres S.L., Espinosa-Cuevas Á., Torre A. (2015). Malnutrition assessed through phase angle and its relation to prognosis in patients with compensated liver cirrhosis: A prospective cohort study. Dig. Liver Dis..

[55] Belarmino G., Gonzalez M.C., Torrinhas R.S., Sala P., Andraus W., D’aLbuquerque L.A.C., Pereira R.M.R., Caparbo V.F., Ravacci G.R., Damiani L. (2017). Phase angle obtained by bioelectrical impedance analysis independently predicts mortality in patients with cirrhosis. World J. Hepatol..

[56] Pagano A.P., Sicchieri J.M.F., Schiavoni I.L., Barbeiro D., Manca C.S., da Silva B.R., Bezerra A.E., Pinto L.C.M., Araújo R.C., Teixeira A.C. (2020). Phase angle as a severity indicator for liver diseases. Nutrition.

[57] Saueressig C., Glasenapp J.H., Luft V.C., Alves F.D., Ferreira P.K., Hammes T.O., Dall’ALba V. (2020). Phase Angle Is an Independent Predictor of 6-Month Mortality in Patients with Decompensated Cirrhosis: A Prospective Cohort Study. Nutr. Clin. Pract..

[58] Shepherd J.A., Ng B.K., Sommer M.J., Heymsfield S.B. (2017). Body composition by DXA. Bone.

[59] Gonzalez M.C., Barbosa-Silva T.G., Heymsfield S.B. (2018). Bioelectrical impedance analysis in the assessment of sarcopenia. Curr. Opin. Clin. Nutr. Metab. Care.

[60] Cruz-Jentoft A.J., Baeyens J.P., Bauer J.M., Boirie Y., Cederholm T., Landi F., Martin F.C., Michel J.-P., Rolland Y., Schneider S.M. (2010). Sarcopenia: European consensus on definition and diagnosis: Report of the European Working Group on Sarcopenia in Older People. Age Ageing.

[61] Alberino F., Gatta A., Amodio P., Merkel C., Di Pascoli L., Boffo G., Caregaro L. (2001). Nutrition and survival in patients with liver cirrhosis. Nutrition.

[62] Sinclair M., Hoermann R., Peterson A., Testro A., Angus P.W., Hey P., Chapman B., Gow P.J. (2019). Use of Dual X-ray Absorptiometry in men with advanced cirrhosis to predict sarcopenia-associated mortality risk. Liver Int..

[63] Eriksen C.S., Kimer N., Suetta C., Møller S. (2021). Arm lean mass determined by dual-energy X-ray absorptiometry is superior to characterize skeletal muscle and predict sarcopenia-related mortality in cirrhosis. Am. J. Physiol. Gastrointest. Liver Physiol..

[64] Santos L.A.A., Lima T.B., Qi X., de Paiva S.A.R., Romeiro F.G. (2021). Refining dual-energy x-ray absorptiometry data to predict mortality among cirrhotic outpatients: A retrospective study. Nutrition.

[65] Belarmino G., Gonzalez M.C., Sala P., Torrinhas R.S., Andraus W., D’aLbuquerque L.A.C., Pereira R.M.R., Caparbo V.F., Ferrioli E., Pfrimer K. (2018). Diagnosing Sarcopenia in Male Patients with Cirrhosis by Dual-Energy X-Ray Absorptiometry Estimates of Appendicular Skeletal Muscle Mass. JPEN J. Parenter. Enteral Nutr..

[66] Mirón Mombiela R., Vucetic J., Rossi F., Tagliafico A.S. (2020). Ultrasound Biomarkers for Sarcopenia: What Can We Tell So Far?. Semin. Musculoskelet. Radiol..

[67] Gödiker J., Schwind L., Jacob T., Böhling N., Groba S.N.R., Kimmann M., Meier J.A., Peiffer K., Trebicka J., Chang J. (2024). Ultrasound-Defined Sarcopenia Independently Predicts Acute Decompensation in Advanced Chronic Liver Disease. J. Cachexia Sarcopenia Muscle.

[68] Becchetti C., Berzigotti A. (2023). Ultrasonography as a diagnostic tool for sarcopenia in patients with cirrhosis: Examining the pros and cons. Eur. J. Intern. Med..

[69] Tandon P., Low G., Mourtzakis M., Zenith L., Myers R.P., Abraldes J.G., Shaheen A.A.M., Qamar H., Mansoor N., Carbonneau M. (2016). A Model to Identify Sarcopenia in Patients With Cirrhosis. Clin. Gastroenterol. Hepatol..

[70] de Luis Roman D., García Almeida J.M., Bellido Guerrero D., Rolo G.G., Martín A., Martín D.P., García-Delgado Y., Guirado-Peláez P., Palmas F., Pérez C.T. (2024). Ultrasound Cut-Off Values for Rectus Femoris for Detecting Sarcopenia in Patients with Nutritional Risk. Nutrients.

[71] Ciocîrlan M., Mănuc M., Diculescu M., Ciocîrlan M. (2019). Is rectus abdominis thickness associated with survival among patients with liver cirrhosis? A prospective cohort study. Sao Paulo Med. J..

[72] Hari A., Berzigotti A., Štabuc B., Caglevič N. (2019). Muscle psoas indices measured by ultrasound in cirrhosis—Preliminary evaluation of sarcopenia assessment and prediction of liver decompensation and mortality. Dig. Liver Dis..

[73] Buchard B., Boirie Y., Cassagnes L., Lamblin G., Coilly A., Abergel A. (2020). Assessment of Malnutrition, Sarcopenia and Frailty 318 in Patients with Cirrhosis: Which Tools Should We Use in Clinical Practice?. Nutrients.

[74] Morgan M.Y., Madden A.M., Soulsby C.T., Morris R.W. (2006). Derivation and validation of a new global method for assessing nutritional status in patients with cirrhosis. Hepatology.

[75] Guglielmi G., Ponti F., Agostini M., Amadori M., Battista G., Bazzocchi A. (2016). The role of DXA in sarcopenia. Aging Clin. Exp. Res..

[76] Saueressig C., Alves B.C., Luft V.C., Anastácio L.R., Santos B.C., Ferreira L.G., Fonseca A.L.F., de Jesus R.P., de Oliveira L.P.M., Boulhosa R.S.d.S.B. (2024). Mid-arm muscle circumference cut-off points in patients with cirrhosis: Low muscle mass related to malnutrition predicts mortality. Nutrition.

[77] Giusto M., Lattanzi B., Albanese C., Galtieri A., Farcomeni A., Giannelli V., Lucidi C., Di Martino M., Catalano C., Merli M. (2015). Sarcopenia in liver cirrhosis: The role of computed tomography scan for the assessment of muscle mass compared with dual-energy X-ray absorptiometry and anthropometry. Eur. J. Gastroenterol. Hepatol..

[78] Nishikawa H., Yoh K., Enomoto H., Iwata Y., Sakai Y., Kishino K., Shimono Y., Ikeda N., Takashima T., Aizawa N. (2020). Calf Circumference as a Useful Predictor of Sarcopenia in Patients With Liver Diseases. In Vivo.

[79] Merli M., Riggio O., Dally L. (1996). Does malnutrition affect survival in cirrhosis? PINC (Policentrica Italiana Nutrizione Cirrosi). Hepatology.

[80] Tapper E.B., Derstine B., Baki J., Su G.L. (2019). Bedside Measures of Frailty and Cognitive Function Correlate with Sarcopenia in Patients with Cirrhosis. Dig. Dis. Sci..

[81] Luengpradidgun L., Chamroonkul N., Sripongpun P., Kaewdech A., Tanutit P., Ina N., Piratvisuth T. (2022). Utility of handgrip strength (HGS) and bioelectrical impedance analysis (BIA) in the diagnosis of sarcopenia in cirrhotic patients. BMC Gastroenterol..

[82] Hanai T., Shiraki M., Imai K., Suetsugu A., Takai K., Moriwaki H., Shimizu M. (2019). Reduced handgrip strength is predictive of poor survival among patients with liver cirrhosis: A sex-stratified analysis. Hepatol. Res..

[83] Short Physical Performance Battery (SPPB) [Internet]. USA: NIH. https://www.nia.nih.gov/research/labs/leps/short-physical-performance-battery-sppb.

[84] Podsiadlo D., Richardson S. (1991). The timed ‘Up & Go’: A test of basic functional mobility for frail elderly persons. J. Am. Geriatr. Soc..

[85] Soto R., Díaz L.A., Rivas V., Fuentes-López E., Zalaquett M., Bruera M.J., González C., Mezzano G., Benítez C. (2021). Frailty and reduced gait speed are independently related to mortality of cirrhotic patients in long-term follow-up. Ann. Hepatol..

[86] Kim G., Kang S.H., Kim M.Y., Baik S.K. (2017). Prognostic value of sarcopenia in patients with liver cirrhosis: A systematic review and meta-analysis. PLoS ONE.

[87] Jiang M., Hua X., Wu M., Wu J., Xu X., Li J., Meng Q. (2024). Longitudinal Changes in Sarcopenia Was Associated with Survival Among Cirrhotic Patients. Front. Nutr..

[88] Kim T.H., Jung Y.K., Yim H.J., Baik J.W., Yim S.Y., Lee Y.-S., Seo Y.S., Kim J.H., Yeon J.E., Byun K.S. (2022). Impacts of muscle mass dynamics on prognosis of outpatients with cirrhosis. Clin. Mol. Hepatol..

[89] Mauro E., Diaz J.M., Garcia-Olveira L., Spina J.C., Savluk L., Zalazar F., Saidman J., De Santibañes M., Pekolj J., De Santibañes E. (2022). Sarcopenia HIBA Score Predicts Sarcopenia and Mortality in Patients on the Liver Transplant Waiting List. Hepatol. Commun..

[90] Ravaioli F., De Maria N., Di Marco L., Pivetti A., Casciola R., Ceraso C., Frassanito G., Pambianco M., Pecchini M., Sicuro C. (2023). From Listing to Recovery: A Review of Nutritional Status Assessment and Management in Liver Transplant Patients. Nutrients.

[91] Christodoulidis G., Tsagkidou K., Bartzi D., Prisacariu I.A., Agko E.S., Koumarelas K.E., Zacharoulis D. (2025). Sarcopenia and Frailty: An In-Depth Analysis of the Pathophysiology and Effect on Liver Transplant Candidates. World J. Hepatol..

[92] Markakis G.E., Lai J.C., Karakousis N.D., Papatheodoridis G.V., Psaltopoulou T., Merli M., Sergentanis T.N., Cholongitas E. (2025). Sarcopenia As a Predictor of Survival and Complications of Patients With Cirrhosis After Liver Transplantation: A Systematic Review and Meta-Analysis. Clin. Transplant..

[93] Ferreira A.P., Machado M.V. (2021). Impact of Pretransplant Frailty and Sarcopenia on the Post-Transplant Prognosis of Patients with Liver Cirrhosis: A Systematic Review. Eur. J. Gastroenterol. Hepatol..

[94] Janssen I., Shepard D.S., Katzmarzyk P.T., Roubenoff R. (2004). The Healthcare Costs of Sarcopenia in the United States. J. Am. Geriatr. Soc..

[95] Goates S., Du K., Arensberg M.B., Gaillard T., Guralnik J., Pereira S.L. (2019). Economic Impact of Hospitalizations in US Adults with Sarcopenia. J. Frailty Aging.

[96] Verified Market Research Sarcopenia Treatment Market Size and Forecast.

[97] Tapper E.B., Zhang P., Garg R., Nault T., Leary K., Krishnamurthy V., Su G.L. (2020). Body Composition Predicts Mortality and Decompensation in Compensated Cirrhosis Patients: A Prospective Cohort Study. JHEP Rep..

[98] Sconfienza L.M. (2019). Sarcopenia: Ultrasound today, smartphones tomorrow?. Eur. Radiol..

[99] Polan D.F., Brady S.L., Kaufman R.A. (2016). Tissue segmentation of computed tomography images using a Random Forest algorithm: A feasibility study. Phys. Med. Biol..

[100] Buckinx F., Landi F., Cesari M., Fielding R.A., Visser M., Engelke K., Maggi S., Dennison E., Al-Daghri N.M., Allepaerts S. (2018). Pitfalls in the measurement of muscle mass: A need for a reference standard. J. Cachexia Sarcopenia Muscle.

[101] Albano D., Messina C., Vitale J., Sconfienza L.M. (2020). Imaging of sarcopenia: Old evidence and new insights. Eur. Radiol..

[102] Rozynek M., Kucybała I., Urbanik A., Wojciechowski W. (2021). Use of Artificial Intelligence in the Imaging of Sarcopenia: A Narrative Review of Current Status and Perspectives. Nutrition.

[103] Bedrikovetski S., Seow W., Kroon H.M., Traeger L., Moore J.W., Sammour T. (2022). Artificial Intelligence for Body Composition and Sarcopenia Evaluation on Computed Tomography: A Systematic Review and Meta-Analysis. Eur. J. Radiol..

